# CAN HEARING AIDS DELAY THE ONSET OF ALZHEIMER’S AND OTHER AGE-RELATED CONDITIONS AMONG ADULTS WITH HEARING LOSS?

**DOI:** 10.1093/geroni/igz038.1385

**Published:** 2019-11-08

**Authors:** Elham Mahmoudi, Tanima Basu, Kenneth Langa, Michael McKee, Phillip zazove, Neil Kamdar

**Affiliations:** 1 University of Michigan, Department of Family Medicine, Ann Arbor, Michigan, United States; 2 University of Michigan, Ann Arbor, Michigan, United States

## Abstract

In this study, we examined the association between hearing aids (HAs) and the onset of Alzheimer’s disease or dementia; depression or anxiety; drug or alcohol disorders; and falls among adults aged 50 and older with hearing loss (HL). We performed a retrospective study of 176,716 adults (50+) with HL diagnoses using a national, insurance claims data (2008-2016). We used Kaplan Meier curves to examine disease-free survival and Cox regression models to examine the risk-adjusted association between HAs and time to diagnosis of 4 age-related/HL-associated conditions within 3 years of HL diagnosis. Large gender and racial/ethnic differences exist in HAs use. Approximately 11.3% of women vs. 14.5% of men used HAs (95% CI Difference: -0.04, -0.03). About 14.1% of Whites (95% CI: 0.14, 0.14) vs. 9.5% of Blacks (95% CI: 0.09, 0.10) and 7.8% of Hispanics (95% CI: 0.07, 0.08) used HAs. The risk-adjusted hazard ratios of being diagnosed with Alzheimer’s disease or dementia, depression or anxiety, drug/alcohol disorders, and injurious falls within 3 years after HL diagnosis, for those who used HA vs. those who did not, were lower by 0.82 (95% CI: 0.76-0.88), 0.92 (95% CI: 0.89-0.95), 0.91 (95% CI:0.80-1.04), and 0.86 (95% CI: 0.81-0.92), respectively. Use of HAs is associated with delayed onset of Alzheimer’s, dementia, depression, anxiety, and injurious falls among adults 50 years of age and older with HL. This is important because HL are increasingly common among older adults and early HL diagnosis and use of HAs may prevent or delay physical and mental decline.

